# Oxycodone versus morphine for cancer pain titration: A systematic review and pharmacoeconomic evaluation

**DOI:** 10.1371/journal.pone.0231763

**Published:** 2020-04-17

**Authors:** Junxiang Zhou, Yixin Wang, Gang Jiang

**Affiliations:** 1 Department of Clinical Pharmcy, Sichuan Cancer Hospital & Institute, Sichuan Cancer Center, School of Medicine, University of Electronic Science and Technology of China, Chengdu, China; 2 Department of Operations Management, Sichuan Cancer Hospital & Institute, Sichuan Cancer Center, School of Medicine, University of Electronic Science and Technology of China, Chengdu, China; Centro di Riferimento Oncologico, ITALY

## Abstract

**Objective:**

To evaluate the efficacy, safety and cost-effectiveness of Oxycodone Hydrochloride Controlled-release Tablets (CR oxycodone) and Morphine Sulfate Sustained-release Tablets (SR morphine) for moderate to severe cancer pain titration.

**Methods:**

Randomized controlled trials meeting the inclusion criteria were searched through Medline, Cochrane Library, Pubmed, EMbase, CNKI,VIP and WanFang database from the data of their establishment to June 2019. The efficacy and safety data were extracted from the included literature. The pain control rate was calculated to eatimate efficacy. Meta-analysis was conducted by Revman5.1.4. A decision tree model was built to simulate cancer pain titration process. The initial dose of CR oxycodone and SR morphine group were 20mg and 30mg respectively. Oral immediate-release morphine was administered to treat break-out pain. The incremental cost-effectiveness ratio was performed with TreeAge Pro 2019.

**Results:**

19 studies (1680 patients)were included in this study. Meta-analysis showed that the pain control rate of CR oxycodone and SR morphine were 86% and 82.98% respectively. The costs of CR oxycodone and SR morphine were $23.27 and $13.31. The incremental cost-effectiveness ratio per unit was approximate $329.76. At the willingness-to-pay threshold of $8836, CR oxycodone was cost-effective, while the corresponding probability of being cost-effective at the willingness-to-pay threshold of $300 was 31.6%. One-way sensitivity analysis confirmed robustness of results.

**Conclusions:**

CR oxycodone could be a cost-effective option compared with SR morphine for moderate to severe cancer pain titration in China, according to the threshold defined by the WHO.

## Introduction

Pain affects cancer patients at all stages from diagnosis to palliative care and is one of the most feared and burdensome symptoms [1,2]. It has a substantial impact on both the clinical and humanistic burden of cancer, particularly in the developing regions of the world [3]. Moderate to severe pain is experienced by more than one third of patients with advanced cancer [4]. Cancer pain is the major factor of influencing the life quality of cancer patients so alleviating pain is of great significance for the treatment of cancer. For the past 30 years cancer pain has been managed according to the World Health Organization (WHO) analgesic ladder [5].

The three-step ladder recommends sequential increases in the strength of analgesia, starting with non-opioid, adding in a weak opioid and finally progressing to a strong opioid until adequate pain relief with minimal adverse drug reaction. In an open-label multicenter randomized control trial, Oxycodone Hydrochloride Controlled-release Tablets (CR oxycodone) and Morphine Sulfate Sustained-release Tablets (SR morphine) were shown to have no differences in tolerability and efficacy as first-line treatment for moderate to sever cancer pain [6]. However, a cochrane review of twenty-three randomized clinical trials indicated that pain relief was significantly better after treatment with SR morphine than CR oxycodone (SMD 0.14, 95% CI 0.01 to 0.27) for cancer pain [7].

In most patients, oral medications should be used initially because they are noninvasive, convenient, and easy to titrate. Opioid-naïve patients experiencing moderate to severe pain should receive rapid titration of oral 5-15mg morphine or equivalent at initial dose. After sixty minutes, efficacy and adverse effects should be reassessed to determine whether to escalate dose by 50%-100% or repeat the same dose [8]. In cancer pain management sustained-release opioids are used for continuous pain as well as immediate-release opioids for break-out pain [9]. The European Association for Palliative Care research network recommend that both of CR oxycodone and SR morphine could be used to titrate in initial treatment of cancer pain [10]. CR oxycodone offers a biphasic absorption pattern which is consist of an initial rapid onset followed by a prolonged phase. In addition, CR oxycodone and SR morphine require twice-daily(every 12hours a day) dosing compared with traditional titration strategy(four times daily), greatly enhancing their administration in cancer pain titration [11].

Most studies comparing oral morphine and oxycodone were conducted in patients who had already a favorable response to opioid treatment [7,12,13], so they could not conclude which one of the two drugs can be considered superior to the other when used as “cancer pain titration” in clinical practice. A cost analysis of CR oxycodone for cancer pain management in Brazil demonstrated that CR oxycodone could lead to a reduction in total costs related to pain treatment in patients with cancer(392.66 BRL per patient), which would lead to resource savings for the payer [14]. Therefore, the costs are another important aspect to impact clinical treatment decision and rational drug use. However, few published data about the percentage of total medical costs associated with cancer attributable to cancer pain titration can be obtained [15]. On this basis, we launched this study aimed at exploratory assessing the efficacy, safety and cost-effectiveness of CR oxycodone and SR morphine applied in the titration of moderate to severe cancer pain.

## Methods

We conducted an incremental cost-effectiveness analysis based on a decision tree for two titration strategies: CR oxycodone group and SR morphine group. We also conducted a meta-analysis of randomized control trials comparing CR oxycodone and SR morphine to obtain efficacy and safety data. The costs data of two group derived from medicine purchasing price of our hospital. Cancer pain was a complication during cancer therapy, so only direct medical costs were considered, excluding the direct non-medical costs (e.g. transportation costs) and indirect cost (e.g. the loss of productivity). All costs were expressed in Chinese yuan (¥), year 2017 values. The costs were translated to dollar at the rate of $1 = ¥6.7518 (as of 2017) [16].

### Model structure and assumptions

**Fig 1:**
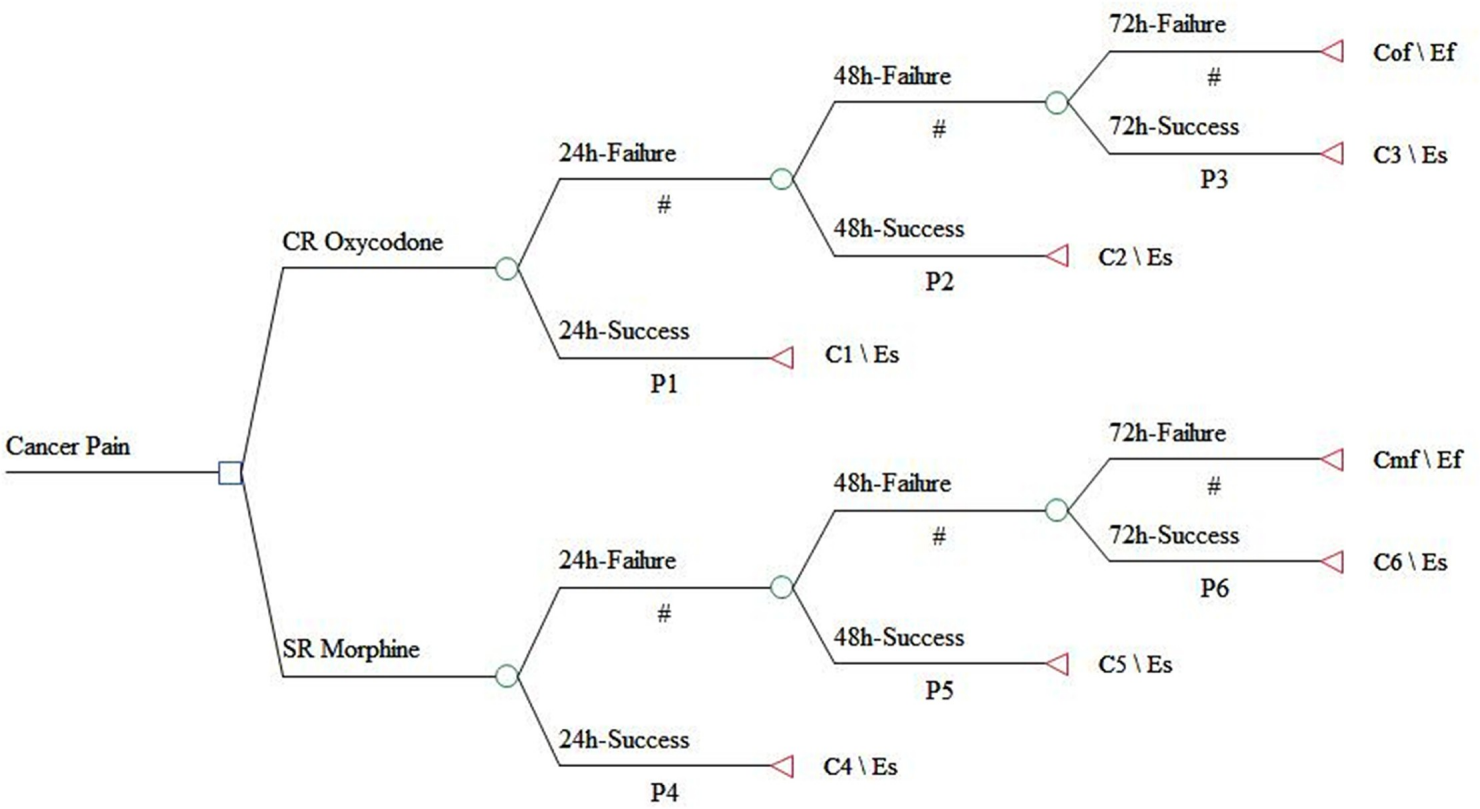
Decision tree model for moderate to severe cancer pain titration.

The structure of the model is shown in Fig 1. This model structure was built upon three assumptions according to NCCN Clinical Practice Guidelines in Oncology of Adult Cancer Pain: (1)The initial dose of CR oxycodone was 20mg. (2)The equivalences of oxycodone compared with morphine based on single-dose was 1:1.5. (3) Oral immediate-release morphine was administered to treat break-out pain [8]. The dose equivalent to 12.5% of total opioid taken in the previous 24h. We developed a decision tree model with a cycle length of 72 hours. The cycle length of 72 hours was chosen because titration should be finished as soon as possible within 48–72 hours recommended by the WHO [17]. This model structure simulated titration of opioid. At the beginning, patients were randomized to either CR oxycodone group or SR morphine group, receiving CR oxycodone 20mg q12h and SR morphine 30mg q12h respectively. If the times of break-out pain exceeded 3 within 24h, dose of CR oxycodone was increased to 40mg q12h as well as SR morphine to 60mg q12h in next 24h. If break-out pain still exceeded 3 times within the second 24h, dose of CR oxycodone was increased to 60mg q12h as well as SR morphine to 90mg q12h in the third 24h. The model cycle was closed whether cancer pain was succeed titration or not after the third 24h. Patients that were titrated successfully in any time can maintain current dose until model cycle closed.

### Study selection and meta-analysis

We searched Medline, Cochrane clinical trials database, Embase, Pubmed, CNKI,VIP and WangFan database from establishment to June 2019. The search strategy included the following keywords: oxycodone, morphine, neoplasm and randomized control trial, combined with free-word retrieval from Mesh and Emtree. Two authors independently finished selection of articles by reading titles, abstracts, full texts or both of them. For some unclear articles, we contacted original authors to identify details by e-mail or telephone. Disagreements were resolved by consensus. The inclusion criteria included: (1) literatures should be designed as randomized controlled trials, (2)the aim population was cancer pain patients, (3) one group used oral CR oxycodone to titrate, another group used oral SR morphine to titrate, (4) Numeric rating scales(NRS) score of cancer pain ≥4, (5) immediate-release morphine was used to treat break-out pain. We excluded studies: (1) cancer pain titration process was not mentioned, (2) immediate-release oxycodone or morphine were used to titrate, (3) patients received ether CR oxycodone or SR morphine combined with other opioids or non-steroidal anti-inflammatory drugs, (4) data on the main outcomes were unavailable.

Data were extracted independently by two reviewers using a standardized collection form. Data from eligible studies were pooled to determine the pain control rate and the rate of adverse drug reaction. The outcomes of pain control rate were divided into 24h pain control rate, 48h pain control rate and 72h pain control rate. The data of adverse drug reaction came from all outcomes of eligible studies reported, such as constipation, nausea, vomiting, pruritus, urinary retentron and anorexia. Meta-analysis was performed by Review Manager5.1.4 which was the Cochrane Collaboration recommendation software. We tested for heterogeneity with significance set at p<0.10 or I^2^>50% using the Chi-squared(χ^2^) test and random effect model was used to calculated [18]. Fixed effect model was used to calculated conversely. The Mantel-Haenszel test was used to estimate risk ratio(RR) and 95% confidence intervals(95%CI). A p-value of less than 0.05 for the test indicated statistically significant heterogeneity.

### Assessment of risk of bias in included studies

According to Cochrane Collaboration Handbook5.1.0 version, quality assessment was made of all included studies, to consider the following questions: (1)Was the assessment to treatment groups truly random? (2)Was allocation adequately concealed? (3)Were those assessing outcomes or blind to the treatment allocation? (4)How were the outcomes considered for people who withdrew and dropout? (5)Were the resemblance between treatment group and control group? (6)Was there any interest conflict bias [18]? The Cochrane Collaboration Handbook criteria are based on the evidence of a strong relationship between the potential bias in the results and methods quality which defined as below: Yes-low risk of bias(adequate); Unclear-moderate risk of bias(some doubt about the result); NO-high risk of bias(inadequate). We contacted authors of all the included studies to acquire details as possible. Publication bias was assessed through testing funnel plot asymmetry which should be interpreted in the light of visual inspection of the funnel plot [18].

### Costs of cancer pain titration and adverse drug reactions

Two types of costs were included in the model: costs associated with cancer pain titration and adverse drug reactions. Costs of cancer pain titration consisted of CR oxycodone, SR morphine and morphine hydrochloride tablets. Following the included paper, we considered only adverse drug reactions that occurred in >1% of each group, such as vomiting, nausea, constipation, pruritus. The probabilities of occurrence of adverse drug reactions were derived from our included studies. The costs of adverse drug reactions were calculated as incidence of adverse drug reactions multiplied by costs of medicine for corresponding events. The resource utilization was estimated based on patient records reviewed at the Sichuan cancer hospital. We adopted phenolphthalein, metoclopramide, diphenhydramine, megestrol and catheterization to manage constipation, emesis, pruritus, anorexia and urinary retentron respectively. The total costs of cancer pain titration and adverse drug reactions were estimated based on official pricelist of National Health Commission of P.R. China. Detailed information of unit costs were shown in Table 1. The costs of cancer pain titration and adverse drug reactions were shown in Table 2. The outcomes of measures for the model were the total costs including CR oxycodone titration costs, SR morphine titration costs and adverse drug reactions costs correspondingly in 2017 Chinese yuan (¥).

**Table 1:** Unit costs used in the model for cancer pain titration and adverse drug reactions.

Resource	Costs(¥)
**Medicine for cancer pain titration**	
Controlled-release Oxycodone 10mg	9.5
Sustained-release Morphine 10mg	4.5
Sustained-release Morphine 30mg	9.9
Morphine Hydrochloride Tablet 5mg	0.865
**Medicine for adverse drug reactions treatment**	
Phenolphthalein Tablet 100mg	0.03
Metoclopramide Tablet 5mg	0.03
Diphenhydramine Hydrochloride Tablet 25mg	0.02
Megestrol Acetate Dispersible Tablet 160mg	10.43
Nursing	12
Urethral catheter per set	16.72

**Table 2:** Costs of titration and adverse drug reactions for cost-effectiveness decision tree.

Resource	Costs(¥)	
	CR oxycodone group	SR morphine group
**Cancer pain titration**		
24h pain control	114.00	59.40
48h pain control	195.19	104.19
72h pain control	243.57	134.37
Pain control failure	259.14	149.94
**Adverse drug reactions treatment**		
Constipation	0.02	0.03
Nausea and vomiting	0.08	0.10
Pruritus	0.02	0.02
Urinary retentron	0.30	0.86
Anorexia	0.64	0.68

### Cost-effectiveness analysis based on a decision tree

The decision-tree-based incremental cost-effectiveness analysis was performed with Decision Tree Software(TreeAge Pro 2019). The events examined in the decision tree were pain controlled rate while receiving opioid titration within model cycle. The probabilities used in the decision tree were determined from the meta-analysis of efficacy and safety. The costs used in the decision tree were those determined in our analysis of the costs of treatment and adverse drug reactions. Incremental cost-effectiveness ratio for the incremental cost per unit gained with each titration strategy were calculated using the formula: ICER = (Co-Cm)/(Eo-Em), where Co and Eo were the costs and pain control rate of CR oxycodone and Cm and Em were the costs and pain control rate of SR morphine.

In the absence of specific utility value for cancer pain titration, we used pain control rate to estimate efficacy. According to NRS score: 0 refers painless; 1–3 refers to mild pain; 4–6 refers to moderate pain; 7–10 refers to severe pain [8]. The criteria of analgesic efficacy evaluation included:(1)complete remission: painless, (2)partial remission: NRS score down to 1–3, (3) mild remission: NRS score down to 4–6, (4)non-remission: stable or incremental NRS score. Pain control rate = (case of complete remission + case of partial remission)/all case.

To examine the stability of the model to alternative input parameters and assumptions, we conducted sensitivity analysis. Here, individual parameters were varied independently with the usual convention being that both a value less than and a value higher than the base-case input parameters were tasted. We summarized the results of sensitivity analysis by examining the effect of changing parameter values or assumptions on the total costs of treatment. Monte Carlo Simulation was conducted to analysis sensitivity of probability [19,20]. Cost-effectiveness acceptability curve and cost-effectiveness scatterplot were displayed.

## Results

### Paper selection and quality assessment

**Fig 2:**
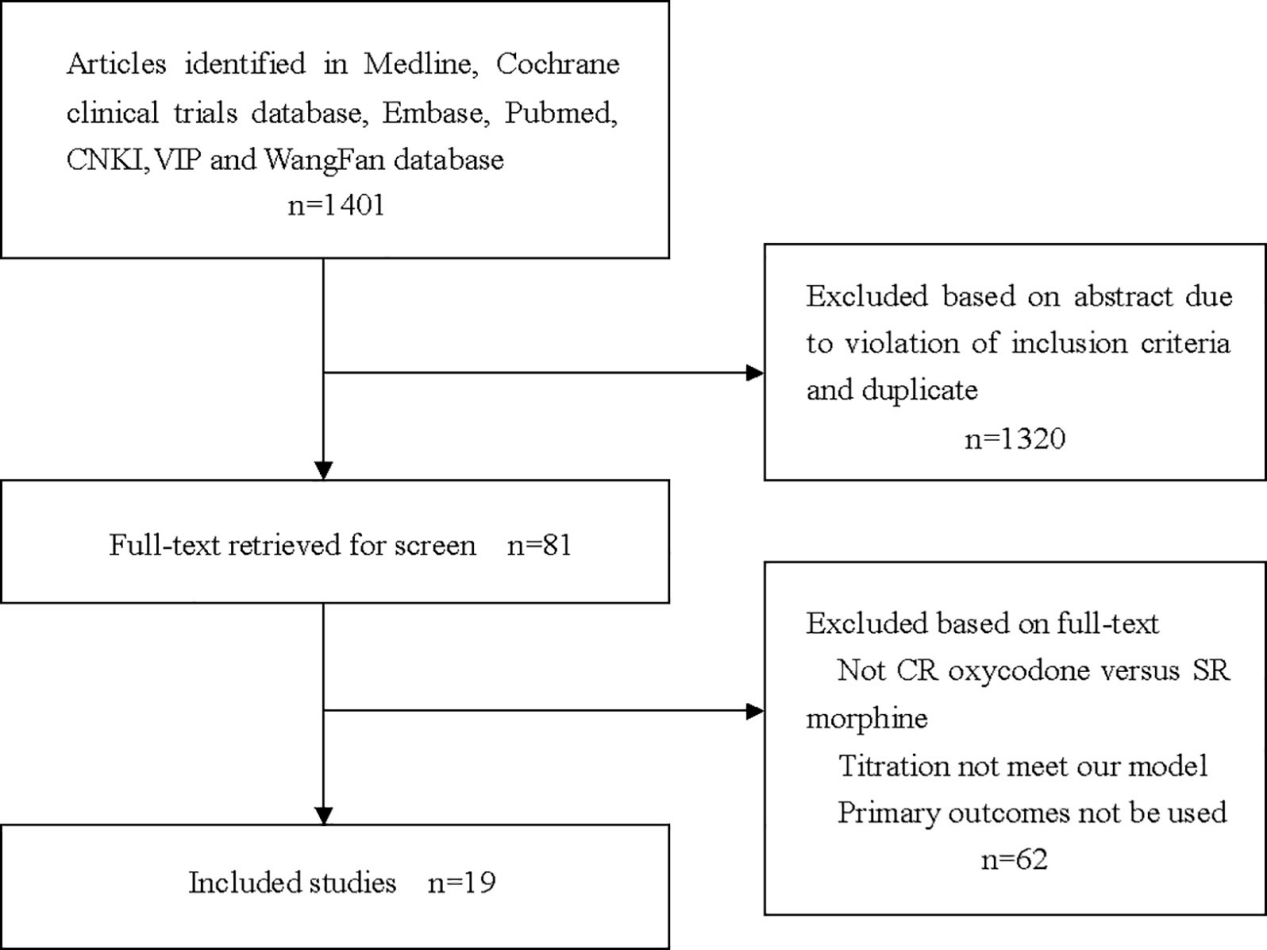
Flow chart of study selection.

**Fig 3:**
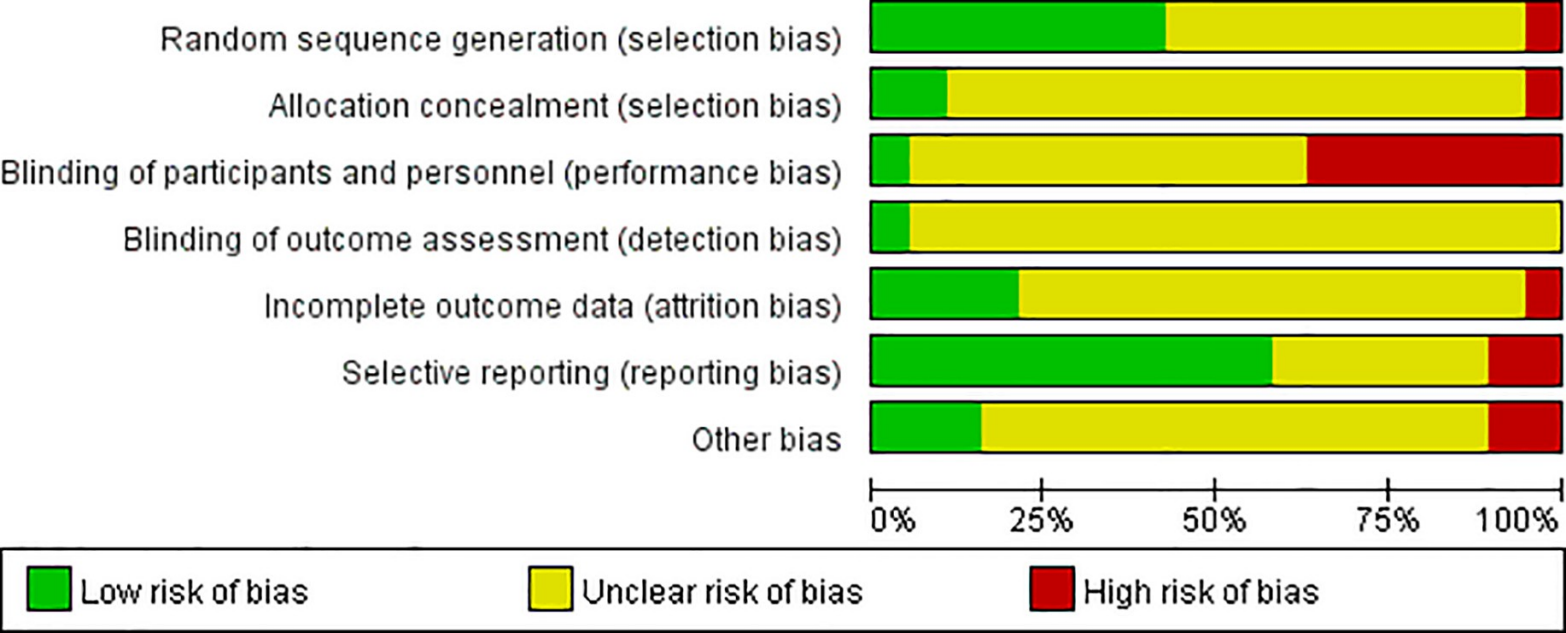
Risk of bias graph.

**Fig 4:**
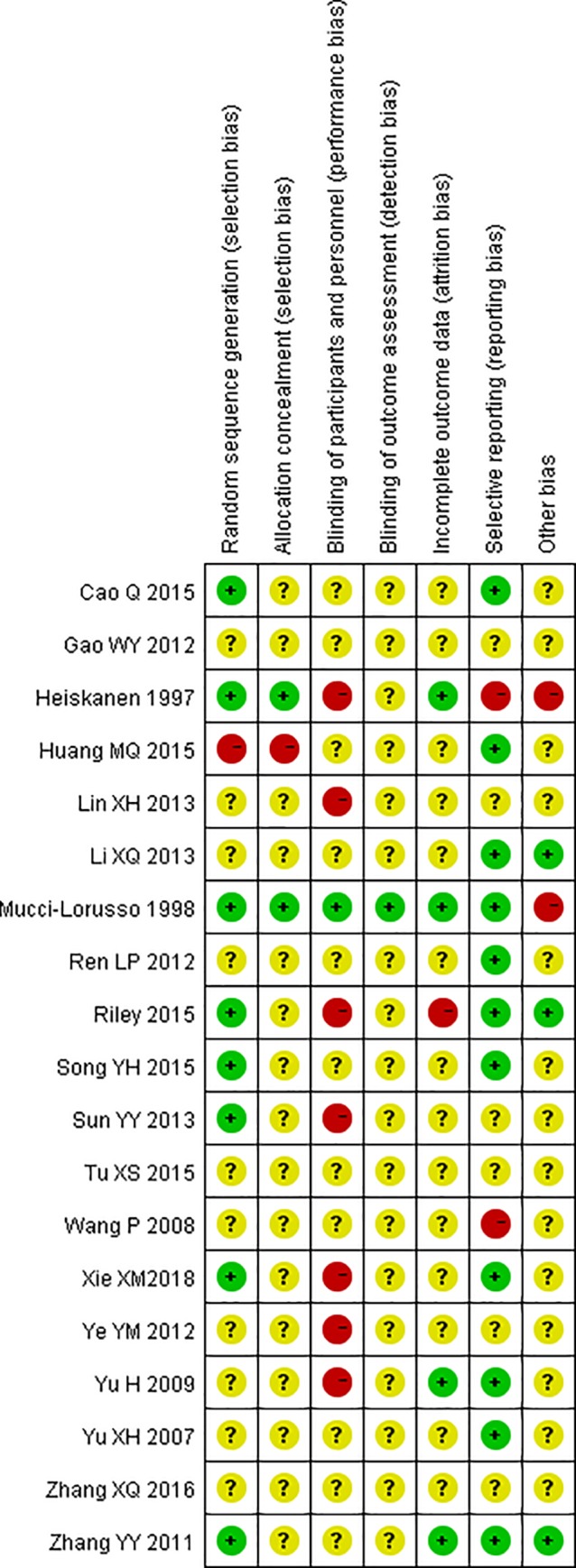
Risk of bias summary.

**Fig 5:**
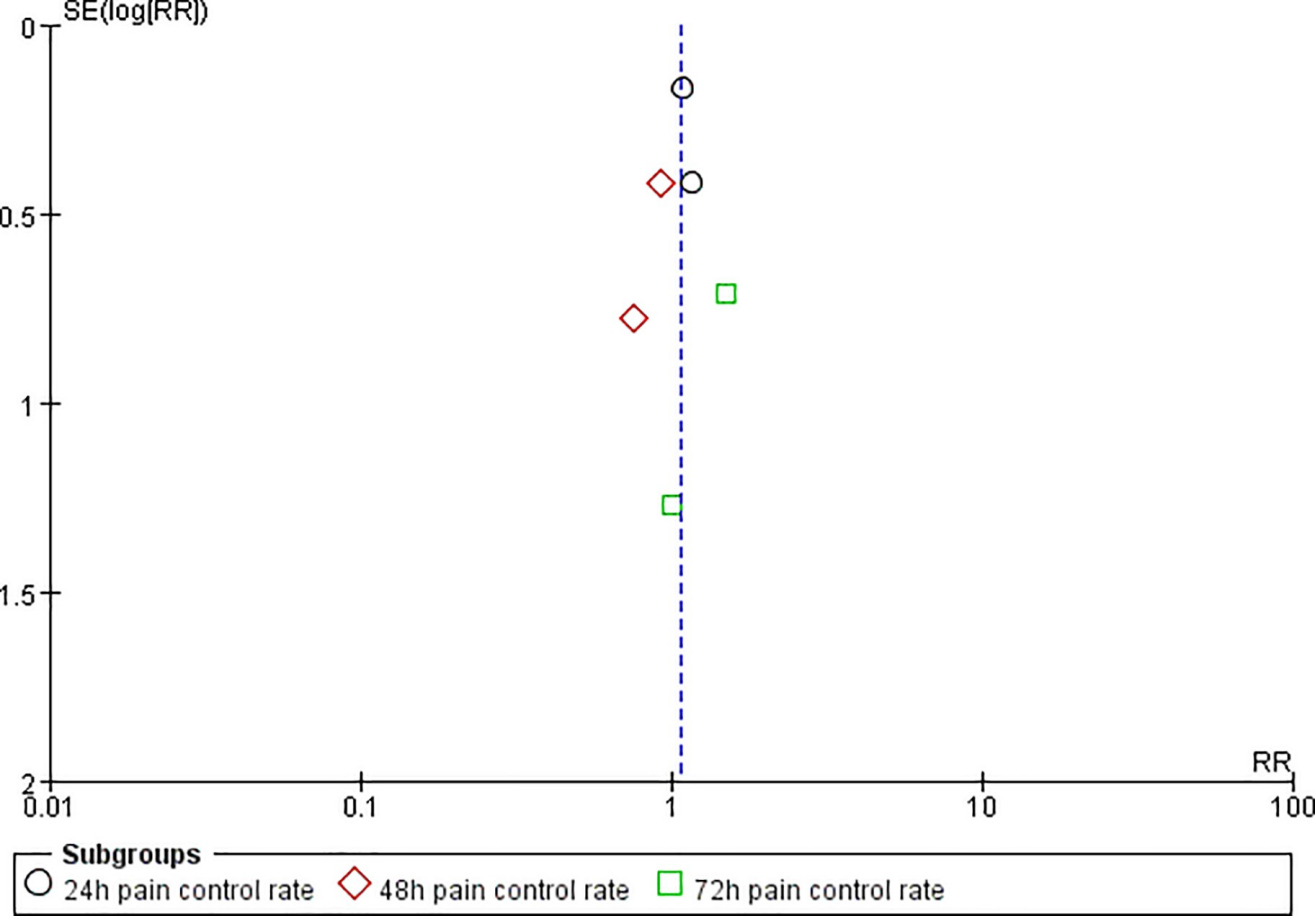
Funnel plot of effective rate.

**Fig 6:**
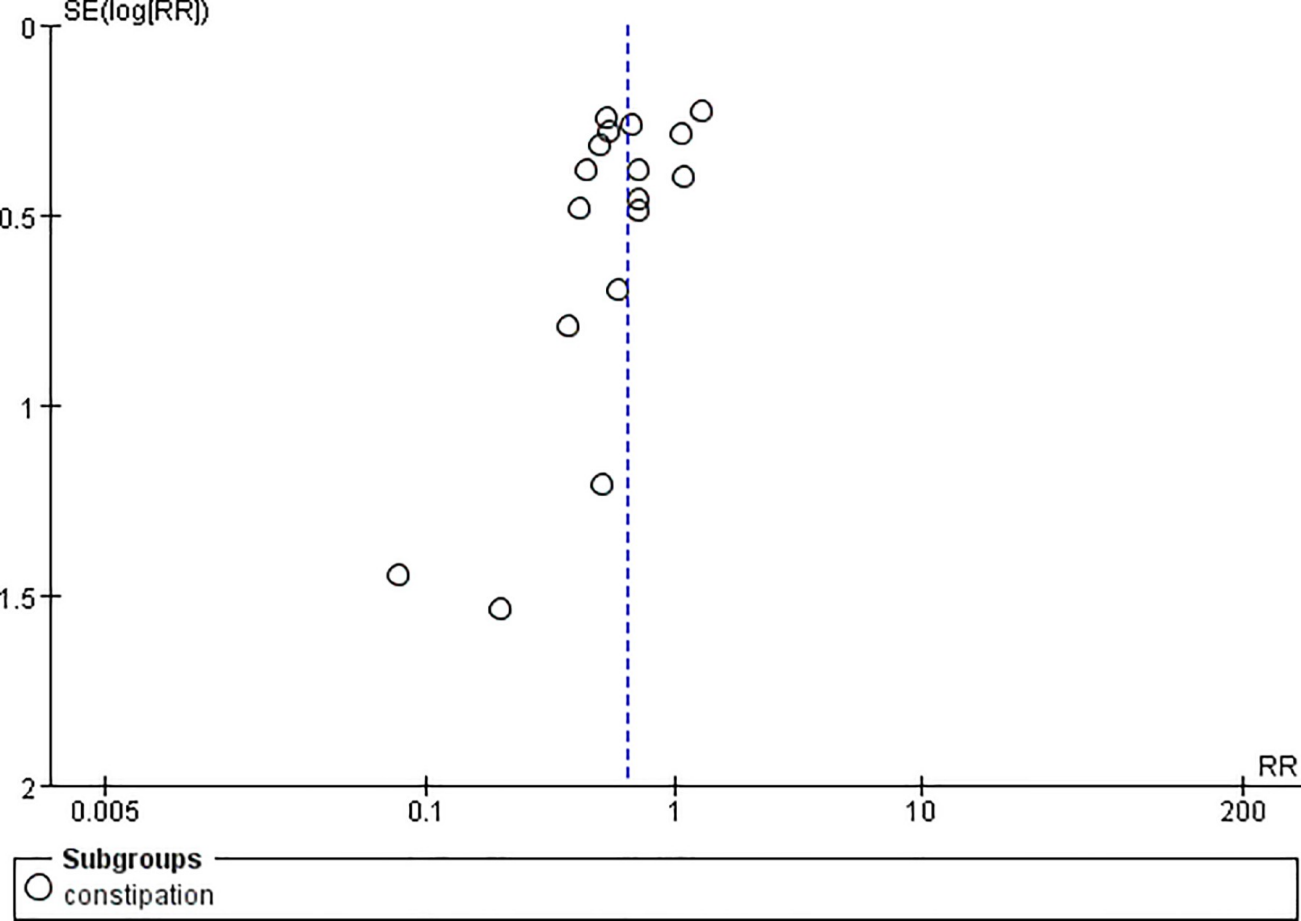
Funnel plot of constipation.

**Fig 7:**
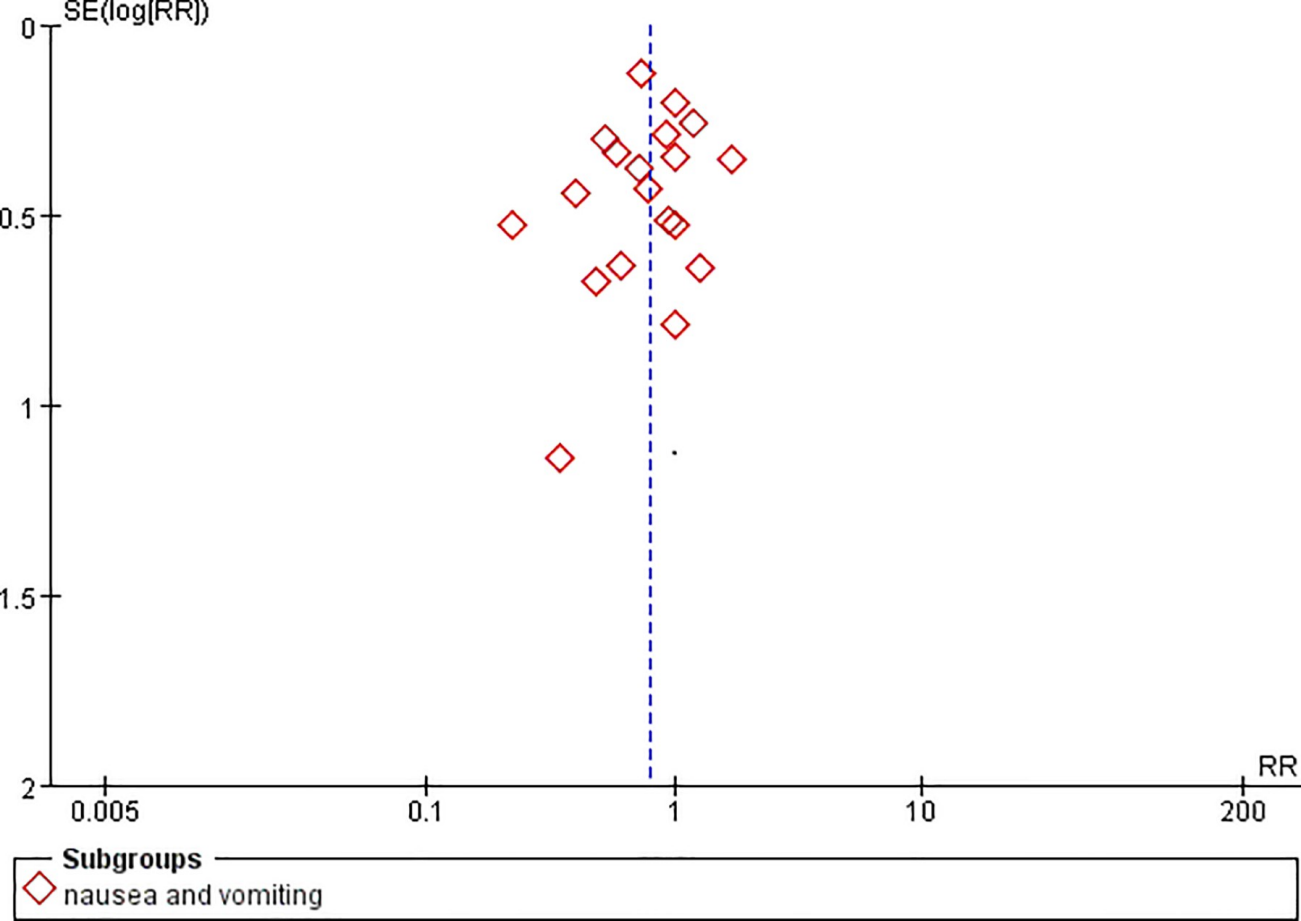
Funnel plot of nausea and vomiting.

**Fig 8:**
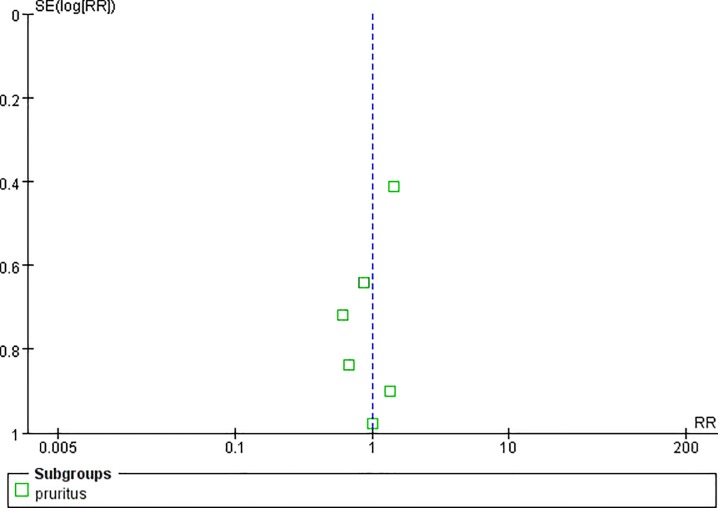
Funnel plot of pruritus.

**Fig 9:**
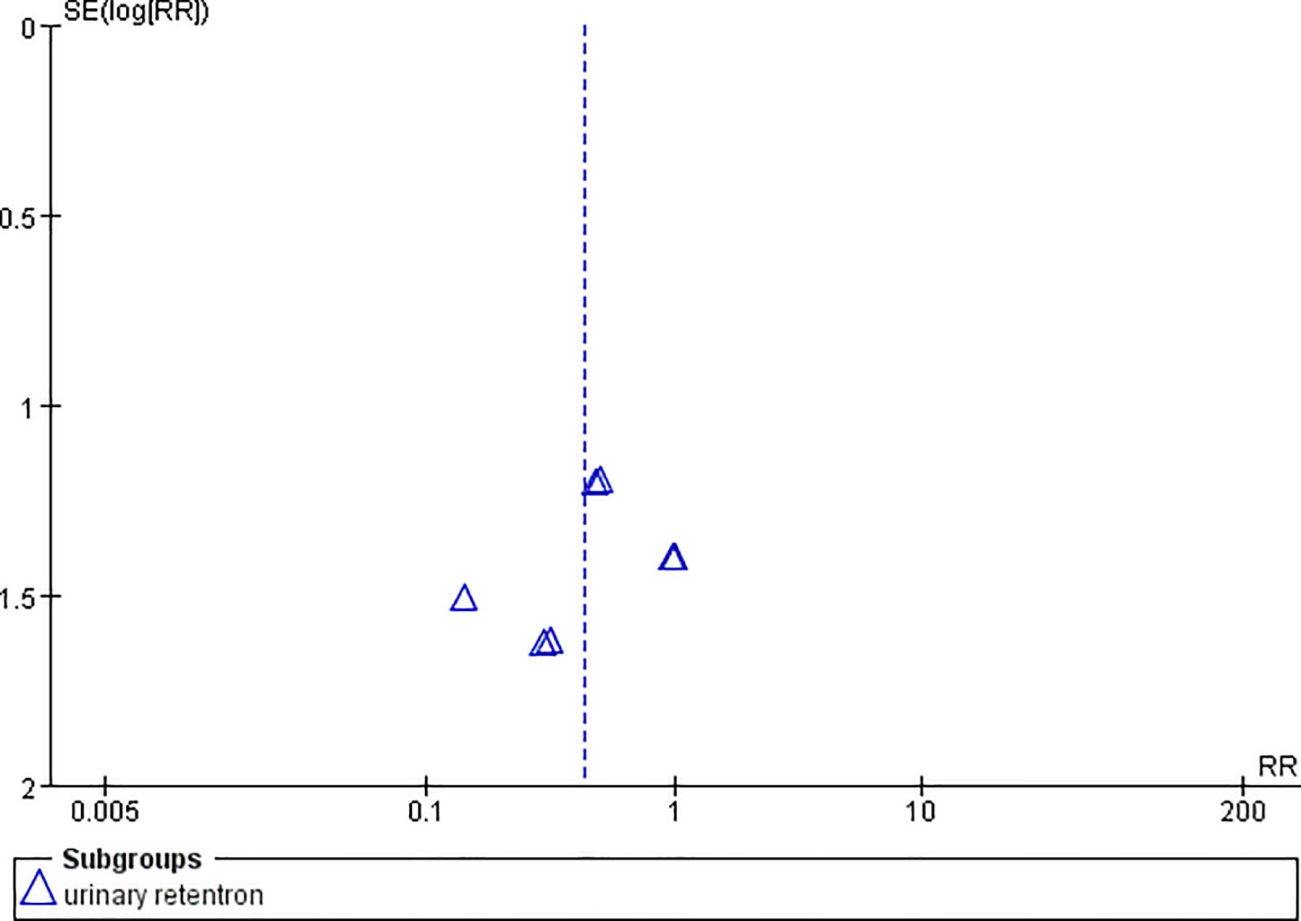
Funnel plot of urinary retentron.

**Fig 10:**
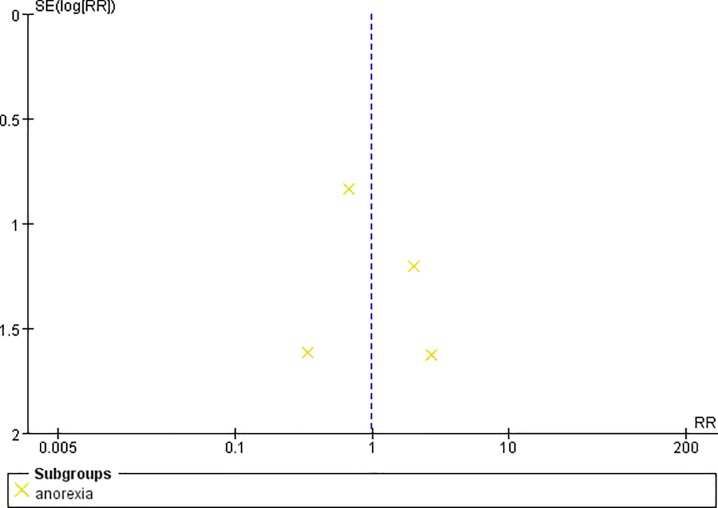
Funnel plot of anorexia.

The search identified 1401 papers; after duplicates were removed,782 papers remained, with 81 papers included for full-text review, and 19 papers (1680 patients)were included in this study [21–39]. Of the excluded papers, 20 papers adopted neither CR oxycodone nor SR morphine as comparison group. The way of cancer pain titration reported in 23 papers could not meet our model, such as subcutaneous administration, drug combination, dosage reversed and cancer pain assessment with visual analog scale. 17 papers did not report original data which our study concern. 2 papers were excluded because the adverse drug reaction data were noted as score. A flow chart describing study selection was presented in Fig 2. The risk of bias graph and the risk of bias summary for the included studies were described in Fig 3, Fig 4. The funnel plots for assessment of publication bias were showed in Figs 5–10.

### Efficacy and safety

Two studies reported the pain control rate (97 patients) [36,38]. There were no significant difference between CR oxycodone and SR morphine for moderate to severe cancer pain titration in 24h(I^2^ = 0, RR = 1.11, 95%CI[0.81,1.51], p = 0.53), 48h(I^2^ = 0, RR = 0.86, 95%CI[0.41,1.80], p = 0.69) and 72h(I^2^ = 0, RR = 1.33, 95%CI[0.40,4.49], p = 0.64). The pain control rate of CR oxycodone for moderate to severe cancer pain titration in 24h, 48h and 72h were 64%, 38.89% and 36.36% respectively. As for SR morphine, the pain control rate were 57.45%, 45% and 27.27% respectively. All of the included studies reported adverse drug reactions. CR oxycodone titration resulted fewer constipation(19.09% vs. 29.63%, p<0.00001) and nausea/vomiting(21.7% vs. 27.4%, p = 0.004) than SR morphine titration, but the probability of pruritus(7.67% vs. 7.79%, p = 0.99), urinary retentron(1.05% vs. 3.01%, p = 0.09) and anorexia(3.07% vs. 3.25%, p = 0.97) were not shown significant difference. (Table 3).

**Table 3:** Probability used for cost-effectiveness decision tree.

Event	Probability(%)		P
	CR oxycodone group	SR morphine group	
**Efficacy**			
24h pain control rate	64	57.45	0.53
48h pain control rate	38.89	45	0.69
72h pain control rate	36.36	27.27	0.64
**Safety**			
Constipation	19.09	29.63	<0.00001
Nausea and vomiting	21.7	27.4	0.005
Pruritus	7.67	7.79	0.99
Urinary retentron	1.05	3.01	0.09
Anorexia	3.07	3.25	0.97

### Total costs

The total costs of CR oxycodone group and SR morphine group were calculated at the events of 24h,48h,72h pain control and pain control failure after 72h separately. For each events, the total costs of CR oxycodone group were higher than SR morphine group. Comparing CR oxycodone with SR morphine, the total costs in 24h, 48h, 72h and pain control failure were $17.04 vs. $9.05, $29.07 vs. $15.68, $36.23 vs. $20.15 and $38.54 vs. $22.46 respectively(Table 4).

**Table 4:** Total costs of treatment for cost-effectiveness decision tree.

Resource	Costs($)	
	CR oxycodone group	SR morphine group
24h pain control	17.04	9.05
48h pain control	29.07	15.68
72h pain control	36.23	20.15
Pain control failure	38.54	22.46

### Cost-effectiveness analysis based on a decision tree

**Fig 11:**
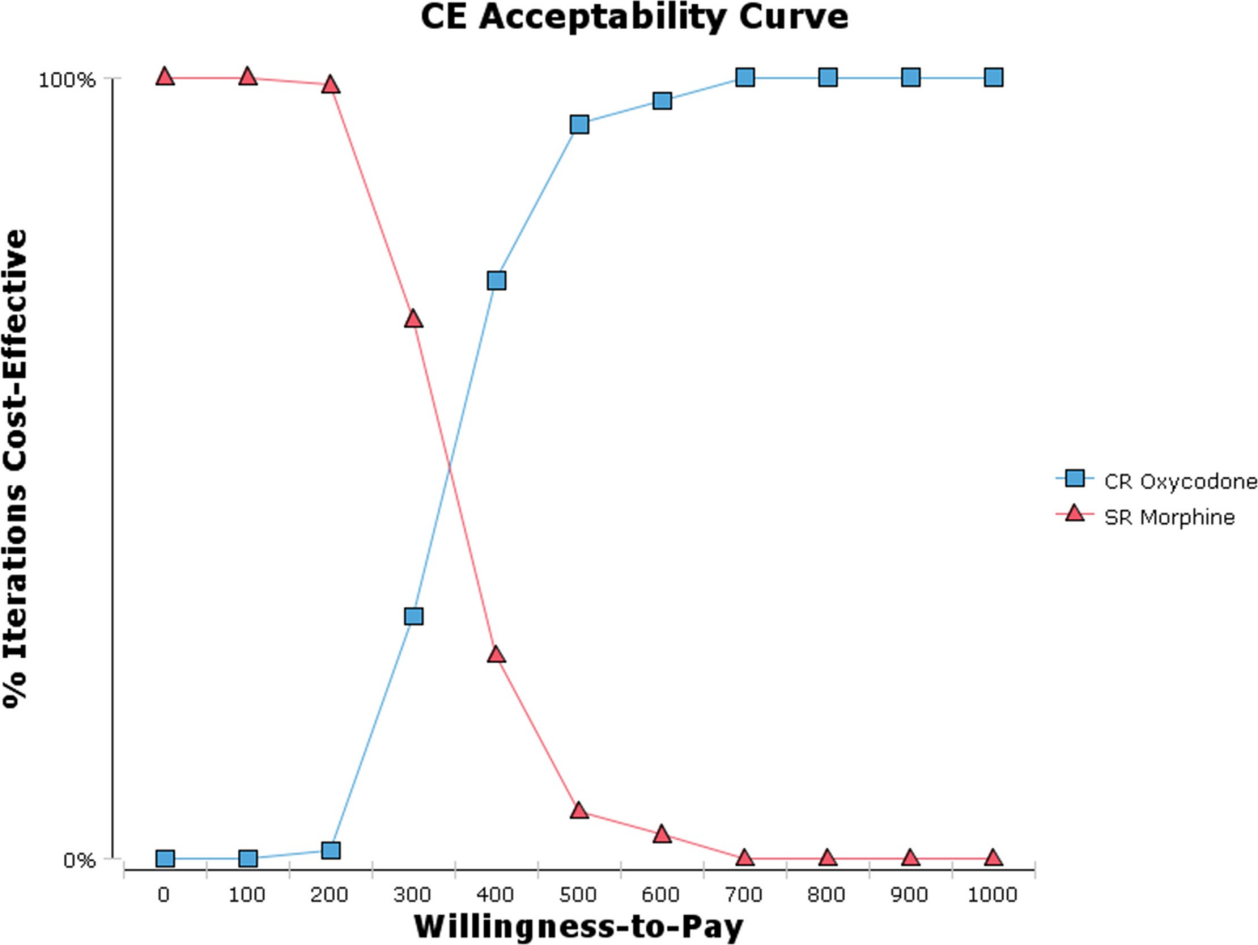
Cost-effectiveness acceptability curve.

**Fig 12:**
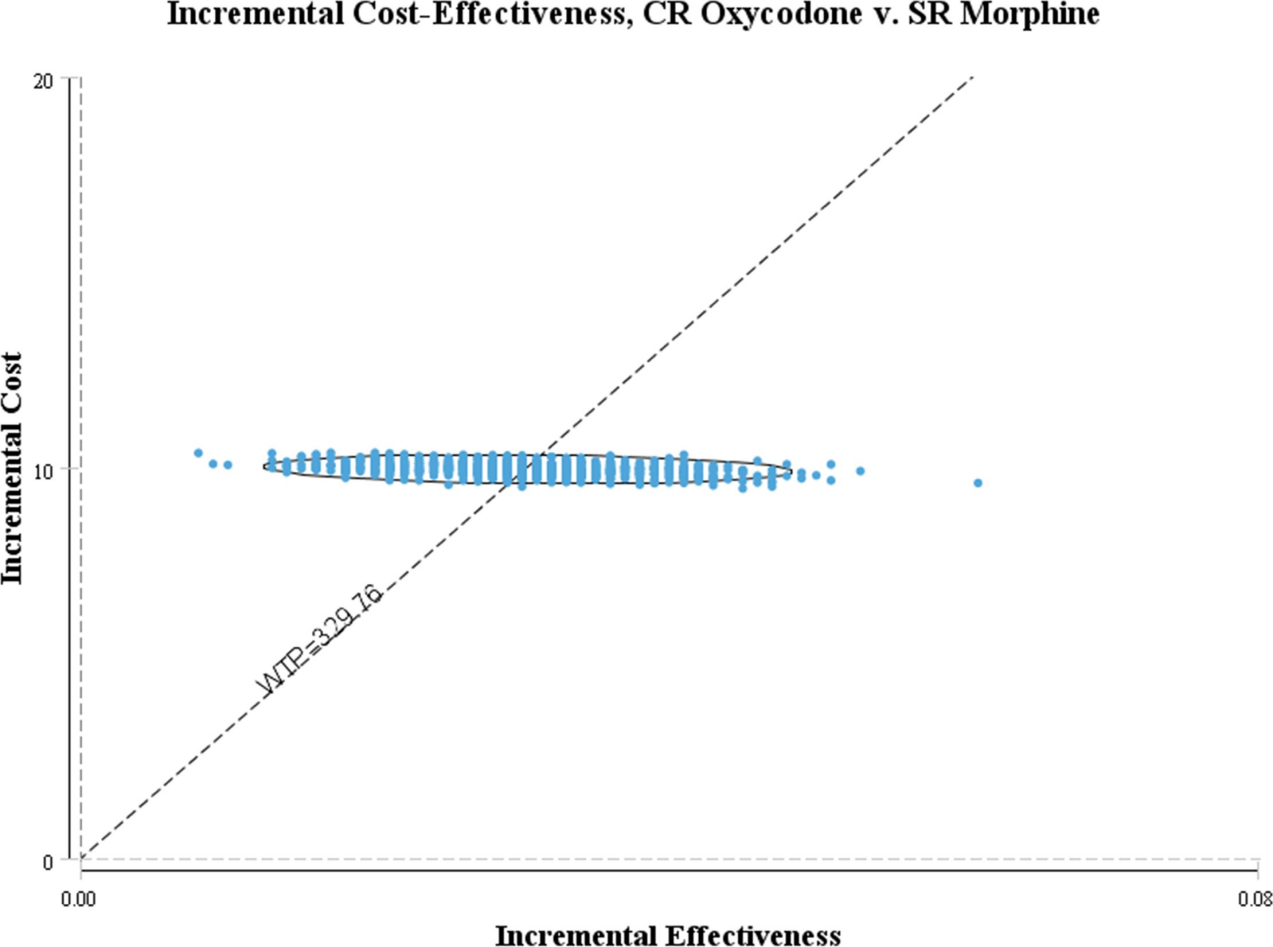
Scatter plot: incremental cost-effectiveness based on 1000 Monte Carlo simulations.

The result of cost-effectiveness analysis showed that the costs per patient for cancer pain titration with CR oxycodone was $23.27, while the costs was $13.31 by using SR morphine. The pain control rate of cancer pain titration were 86.00% and 82.98% by using CR oxycodone and SR morphine respectively (Table 5). The ICER value was $329.76 per unit. Thousand samples were calculated by using Monte Carlo Simulation for our established decision tree. The simulation running results were shown as cost-effectiveness acceptability curve (Fig 11) and cost-effectiveness scatter plot (Fig 12). The probability of ICER per unit below $300, $400, $500 and $600 were 31.6%, 73.8%, 90.9% and 96.7% respectively.

**Table 5:** Cost-effectiveness analysis based on a decision tree.

Titration strategy	Costs per patient for cancer pain titration($)	Pain control rate of cancer pain titration
CR oxycodone	23.27	0.8600
SR morphine	13.31	0.8298

1$ = 6.7518¥

Alternative assumptions and parameters were investigated in sensitivity analysis. Three titration strategies were used to change the research assumption to test the stability of the results. If the initial titration dose of CR oxycodone is set at 10mg, the costs of CR oxycodone will reduced to a similar level of SR morphine (Table 6). The ICER per unit of CR oxycodone compared with SR morphine were $348.05, $0.63 and $0.51 at titration dose of CR oxycodone 20mg-40mg-80mg strategy, 10mg-20mg-30mg strategy and 10mg-20mg-40mg strategy respectively. With regard to changing of costs, the one-way sensitivity analysis showed that an increasing of 100% in CR oxycodone acquisition costs, the ICER per unit increased to $1064.13. Other alternative parameters were used that increased of 15% in CR oxycodone acquisition costs, reduced of 15% in SR morphine acquisition costs, increased of 100% in adverse drug reactions treatment costs and reduced of 50% in adverse drug reaction treatment costs. The above mentioned variation of input parameter displayed minimal impact on the ICER.

**Table 6:** Incremental cost-effectiveness ratio of CR oxycodone compared with SR morphine for each variation of input parameter in sensitivity analysis.

Variable	Costs($)		ICER per unit ($)
	CR oxycodone group	SR morphine group	
**Titration strategy**			
Titration dose of CR oxycodone 20mg-40mg-80mg	24.51	14.00	348.05
Titration dose of CR oxycodone 10mg-20mg-30mg	11.77	11.75	0.63
Titration dose of CR oxycodone 10mg-20mg-40mg	12.39	12.37	0.51
**Drug-acquisition costs(**$**)**			
CR oxycodone +100%	45.45	13.31	1064.13
CR oxycodone +15%	26.59	13.31	439.82
SR morphine -15%	23.27	11.51	389.27
Adverse drug reactions treatment +100%	23.43	13.56	326.78
Adverse drug reactions treatment -50%	23.19	13.18	331.33

## Discussion

This is the first study to evaluate the economic of CR oxycodone compared with SR morphine in the treatment of cancer pain titration based on a decision tree model. Decision tree model increasingly use cost-effectiveness analysis to inform decision making on competing health care interventions. Economic models facilitate these analysis by providing a framework to combine information from different sources and enable probabilistic estimations. Our cost-effectiveness analysis based on a decision tree model predicted the benefits of CR oxycodone compared with SR morphine for moderate to severe cancer pain titration. The relative costs of cancer pain therapies has become an increasingly important issue in recent years due to growing concerns about the rising costs of health care and the lack of data demonstrating the superiority of one agent over another.

In our modeling analysis, CR oxycodone had higher efficiency than SR morphine in the treatment of moderate to severe cancer pain titration(86% vs. 82.98%), while had more expensive costs($23.27 vs. $13.31). The willingness-to-pay(WTP) threshold has not been officially defined in China, the general consensus is to be set at 1 gross domestic product(GDP) per capita, which is defined by the WHO [40]. Our model showed that CR oxycodone could be considered cost-effective compared with SR morphine in terms of ICER per unit below the 1GDP of P.R. China in 2017($8836) [16]. Due to higher costs, CR oxycodone could not be deemed to be cost-saving. The probability sensitivity analysis demonstrated that the probability of CR oxycodone being cost-effective at the WTP threshold of $300 and $600was 31.6% and 96.7% respectively. Considering the heavy economic burden for patients associated with cancer treatment especially in antineoplastic drugs, the WTP of cancer pain titration may far less than 1GDP in developing country. SR morphine would be still a good choice for patient in financial straits. As a result of unique drug release technology of CR oxycodone, CR oxycodone may be better than SR morphine in cancer pain titration, but there was no evidence to support it. At our knowledge, this is the first pharmacoeconomic study to support that perspective. The economic model established in this study can be generalized to other countries for cost-effectiveness analysis of opioid titration by inputting local data.

For one-way sensitivity analysis, we further assumed that titration strategy would change in real world treatment. Three different titration strategies were tested in the model: (1) Day1 (CR oxycodone20mg, SR morphine30mg)-Day2 (CR oxycodone40mg, SR morphine60mg)-Day3(CR oxycodone80mg, SR morphine120mg), (2) Day1 (CR oxycodone10mg, SR morphine20mg)-Day2 (CR oxycodone20mg, SR morphine40mg)-Day3(CR oxycodone30mg, SR morphine60mg), (3) Day1 (CR oxycodone10mg, SR morphine20mg)-Day2 (CR oxycodone20mg, SR morphine40mg) -Day3(CR oxycodone40mg, SR morphine80mg). Despite this, CR oxycodone remained cost-effective. In addition, drug-acquisition costs was another crucial factor. Whether CR oxycodone price raised by 100%,15% or SR morphine price decreased by 15%, the ICER per unit still below the 1GDP of P.R. China in 2017($8836). Due to low costs of drugs to treat opioid adverse reactions in China, it had little impact on outcomes in spite of increasing 100% or decreasing 50% the costs of adverse drug reactions. Hence, one-way sensitivity analysis demonstrated CR oxycodone was a cost-effective choice.

It is true that there are methodological problems and a plethora of extraneous variables that influence the reliability and validity of studies conducted in pharmacoeconomic area [3]. Some limitations of our study need to be taken into account when interpreting the results. First, the use of efficacy data from two randomized control trials [36,38] may limit the ability to generalize the findings of this analysis to a broader population, and more up-to-date clinical data may be available. Patients with cancer pain often receive concurrent chemotherapy or radiotherapy, which also have several side effects that can interfere with observation results of opioid adverse drug reactions. Most of the included studies did not describe whether patients received chemotherapy or radiotherapy during the cancer pain titration. In addition, the included studies in our analysis did not have exactly the same titration strategy and course of treatment that may effect on adverse drug reaction rate and costs estimating. To account for such differences, we conducted several sensitivity analysis. We changed our assumptions of titration strategy to another three common modes and found CR oxycodone remained cost-effective in these conditions. These titration strategies were discussed with clinical experts and were considered reasonable.

Furthermore, the drug-acquisition costs of moderate to severe cancer pain titration were calculated. Due to the cancer pain was a complication in the cancer therapy, the cancer pain titration was implemented in conjunction with chemotherapy or radiotherapy. The other hospitalization costs such as bed fee, chemotherapy fee, radiotherapy fee and indirect costs were not included in our analysis. It is difficult to accurately quantify these costs especially in developing countries where there are limited research and data [3]. Considering the influence of drug price on results, we attempted to ascertain the impact of parameter variation via alternatives in a one-way sensitivity analysis. In all the alternative scenarios conducted, the results were stable and consistent with base-case analysis. In Mexico, the costs of CR oxycodone was $57702.34 for 1-year treatment of cancer pain [41]. Two studies with respect to chronic pain using a 1-year time horizon reported the costs of CR oxycodone were £3656 and $1426.52 in the UK and Canada separately [42,43]. In our study, the cost-effectiveness was calculated based on the first three days of cancer pain titration. So the costs were much lower than the reported in previous literatures.

Finally, there are intrinsic biases that may influence the results. Potential bias could arise from study heterogeneity, such as in study population and study designs. Only three clinical studies [23,27,29] were conducted outside China, so the risk of publication bias having an effect on our results can not be ruled out. Most of the studies had problems of absence of clear descriptions of randomization, allocation concealment and blinding during clinical trial implementation. Two studies were funded [23,27] by pharmaceutical company. Optimal study design of cancer pain titration should be highlighted to provide better evidence for clinical decision.

## Conclusion

In conclusion, our results indicate that, from the People’s Republic of China perspective, oxycodone hydrochloride controlled-release tablets is cost-effective compared with morphine sulfate sustained-release tablets, and should be an optimization choice for moderate to severe cancer pain titration. Sensitivity analysis shows the results to be reasonably insensitive to variability in key assumptions and variables. The model that we have presented is compliant with the current health economic guidelines. Future studies using real-world data are required to confirm our findings.

## Supporting information

S1 Checklist (DOC)

S1 File (DOC)
