# Supplementary material for: Oxycodone versus morphine for cancer pain titration: A systematic review and pharmacoeconomic evaluation
Source: PLoS One. 2020 Apr 17;15(4):e0231763. doi: 10.1371/journal.pone.0231763 (PMC7164642; doi:10.1371/journal.pone.0231763)
Supplement: S1 File — (DOC) [file pone.0231763.s002.doc]

Pubmed:

#1 randomized controlled trial[Publication Type]

#2 controlled clinical trial[Publication Type]

#3 randomized[Title/Abstract]

#4 placebo[Title/Abstract]

#5 randomly[Title/Abstract]

#6 trial[Title/Abstract]

#7 groups[Title/Abstract]

#8 drug therapy[MeSH Subheading]

#9 or#1-#8

#10 oxycodone[MeSH Terms]

#11 oxycodone[Text Word]

#12 Oxycone[Text Word]

#13 Dihydrone[Text Word]

#14 Oxycodeinon[Text Word]

#15 Eucodal[Text Word]

#16 Theocodin[Text Word]

#17 oxycodone hydrochloride[Text Word]

#18 Oxycontin[Text Word]

#19 Pancodine[Text Word]

#20 Dinarkon[Text Word]

#21 Oxiconum[Text Word]

#22 or#10-#21

#23 morphine[MeSH Terms]

#24 morphine[Text Word]

#25 morphia[Text Word]

#26 MS Contin[Text Word]

#27 Oramorph SR[Text Word]

#28 Duramorph[Text Word]

#29 SDZ 202 250[Text Word]

#30 or#23-#29

#31 neoplasms[MeSH Terms]

#32 neoplasm*[Text Word]

#33 Tumor*[Text Word]

#34 Cancer*[Text Word]

#35 Malignancy*[Text Word]

#36 Malignant[Text Word]

#37 or#31-#36

#38 #9 and #22 and #30 and #37

Embase:

#1 'controlled clinical trial'/exp

#2 'double blind procedure'/exp

#3 'single blind procedure'/exp

#4 'crossover procedure'/exp

#5 'prospective study'/exp

#6 'comparative study'/exp

#7 'randomization'/exp

#8 'placebo'/exp

#9 blind*:ab,ti

#10 random*:ab,ti

#11 control*:ab,ti

#12 placebo*:ab,ti

#13 or/#1~#12

#14 ‘oxycodone’ /syn AND [embase]/lim

#15 ‘morphine’/syn AND [embase]/lim

#16 ‘neoplasm’ /syn AND [embase]/lim

#17 #13 and #14 and #15 and #16
